# The Combined Effects of Patient Activation and Relational Aspects on the Quality of Life in Atrial Fibrillation Patients

**DOI:** 10.3389/fpsyg.2021.761149

**Published:** 2021-11-18

**Authors:** Jie Wang, Qin Wang, Zhipeng Bao, Yuanyuan Peng, Shenxinyu Liu, Tianxi Yu, Lin Wang, Gang Yang, Min Gao, Guozhen Sun

**Affiliations:** ^1^School of Nursing, Nanjing Medical University, Nanjing, China; ^2^Department of Cardiology, The First Affiliated Hospital of Nanjing Medical University, Nanjing, China

**Keywords:** patient activation, quality of life, atrial fibrillation, cardiac rehabilitation, cross-sectional study

## Abstract

**Objective:** This study aims to explore the influence of patient activation (PA) and relational aspects on the quality of life (QoL) in patients with Atrial Fibrillation (AF) for developing measures to improve PA and QoL.

**Methods:** A cross-sectional study was undertaken in 2021 among 190 AF patients in Nanjing, China. Research instruments included a self-designed social-demographic characteristics scale, the Patient Activation Measure (PAM), the Atrial Fibrillation Effect on Quality of Life (AFEQT). The data analysis was performed using IBM SPSS 25.0. Spearman correlation analysis, multiple linear regression analysis, and Wilcoxon rank-sum tests were used to assess the association accordingly.

**Results:** The average AFEQT score for the 190 AF patients was 69.32 ± 14.52. The distribution of activation Levels 1, 2, 3, and 4, were where 4.7, 34.2, 47.4, and 13.7%, respectively. The multiple linear regression analysis revealed that patient activation, work status, and cardiac rehabilitation of AF patients predicted AF-related QoL (β = 0.270, −0.205, and 0.183, respectively; all *P* < 0.05). The influences of PA level on subdimensions of AF-related QoL were as follows: symptoms, daily activities and treatment concern.

**Conclusion:** The level of QoL of patients with AF was moderate. Higher levels of patient activation in those with AF were associated with milder symptoms, more positive daily activities and fewer treatment concern. Based on our findings, we suggest that healthcare personnel should encourage AF patients to take active participation in cardiac rehabilitation, disease self-management and foster progression of PA level. Future research is warranted to develop tailor-made interventions aimed at the activation level.

## Introduction

Prevalent of chronic illnesses are increasing rapidly due to population aging. The diminished quality of life in elderly population has fostered the general concern and leads to an increasing demand for optimizing care and health-related quality of life (QoL) (Jiang et al., 2017; Thomas et al., 2020; Jing et al., 2021). Atrial fibrillation (AF) is the most common sustained arrhythmia and usually not life-threatening. It is a significant public health issue among the increasingly elderly population which seriously affects the quality of life. Currently, the estimated AF prevalence in adults is between 2 and 4% worldwide (Hindricks et al., 2021). In a large national representative, community-based study, the weighted AF prevalence for age >45 years old was 1.8% in China (Du et al., 2021). Kao et al. (2010) showed that quality of life is an independent risk factor for rehospitalization rate and mortality in AF patients. In 2000, the Working Group on Atrial Fibrillation of the European Society of Cardiology emphasized that clinicians should include health-related QoL as important indicators of assessment of treatment outcomes (Camm et al., 2012).

About one-third of AF patients had no obvious clinical symptoms (Dilaveris and Kennedy, 2017), which often be neglected regarding the prevention of heart failure and thromboembolism. AF symptoms include palpitations, fatigue, dizziness, dyspnea, and presyncope. Reoccurred episodes of these symptom greatly increase the psychological burden and reduce the QoL. Previous studies have shown that AF patients had a worse QoL regarding different subdimensions assessed by Atrial Fibrillation Effect on Quality-of-Life (AFEQT) than healthy individuals (Wokhlu et al., 2010). Furthermore, symptomatic AF patients demonstrated a significant lower AFEQT total scores than asymptomatic patients (Spertus et al., 2011). However, subclinical atrial tachyarrhythmias, including asymptomatic AF, were shown to have a higher prevalence of stroke and a great impact on QoL (Kupper et al., 2013). Moreover, AF reduces cardiac output and function reserve, which also results in a decrease in QoL (Watanabe et al., 2012).

Patient activation (PA) refers to an individual’s ability to understand their role in managing their own health and having the knowledge, skills, and confidence to do so (Hibbard et al., 2004). Patient’s engagement in health care is a fundamental component of quality healthcare and improves therapy efficacy and prognosis (Karim et al., 2018). In recent years, a growing evidence indicates that higher activation contributes to the better self-management, shorter hospital stay, less medical costs and improvement in functional status. Participation in medical management might decrease medical errors and adverse events by improving patient compliance and realize self-administration.

Recent researches about PA in patients with chronic disease were focused on three aspects–first, activation level and its influencing factors, such as Chen et al. (2020) explored the association of PA and demographic(education, monthly income and illness severity, etc.) in heart failure patients. Second, the relationship between activation and self-management. More targeted interventions have been developed to strengthen and maintain self-management behaviors by early identification of the activation level. A randomized controlled trial indicated that individualized telephone health coaching might improve treatment adherence, improve health outcomes, and decrease health resource utilization (Oddone et al., 2018). Third, the relationship between activation and health outcomes. Milo et al. (2021) reported that activation could predict glycemic control and lower low-density lipoprotein. However, little is known about patient activation in AF patients. By integrating the knowledge of the activation level and its relationship to QoL in AF patients are important for promoting patient autonomy and shared decision making.

Therefore, the purposes of this study were (1) to investigate the PA and QoL of AF patients, (2) to assess how PA and sociodemographic characteristics contribute to AF-related QoL, and (3) to analyze how the PA level affect the QoL and its subdimensions.

## Materials and Methods

### Design, Sample, and Settings

The present study is a cross-sectional study. The convenience sampling was employed to recruit participants from the Department of Cardiology at the First Affiliated Hospital of Nanjing Medical University. The inclusion criteria were (1) diagnosed with AF according to ECG-documented and symptoms; (2) patients ≥18 years of age; (3) able to provide oral and written informed consent. Patients were excluded: (1) patients with cognitive impairment or severe psychiatric disorders; (2) patients with any severe diseases, with life-threatening complications, such as malignancy, multiple organ dysfunction.

### Measurements

#### Sociodemographic Variables

The collected data included age, gender, education level, marital status, work status, health insurance, monthly income, type of AF, the number of radiofrequency ablation, and participation in cardiac rehabilitation (CR) or not.

#### Patient Activation

Patient activation was measured using the Patient Activation Measure developed by Hibbard (Hibbard et al., 2004). The scale is an abbreviated version of PAM-22 with 13 items (Hibbard et al., 2005). The questionnaire is composed of four dimensions: cognition, skill, action, and belief. The items are measured with a Likert scale of five points (one-strongly disagree, five-not applicable). The score is calculated by summing up the raw scores and mapping up the sum onto a scale of 0–100. The PAM13 score is higher, the patient activation level is higher. PA is divided into four stages according to the score: level 1 (patients are at a loss and cannot play an active role in the process of disease diagnosis and treatment, score <47.0), level 2 (patients are short of confidence and knowledge on disease self-management, 47.1∼55.1), level 3 (patients start taking an active role in their treatment, but still lack enough confidence and skills to support their actions, 55.2∼67.0), and level 4 (patients bear their disease with confidence and take actions, but cannot stick to their behaviors in a stress situation, score ≥67.1). The questionnaire has been validated for multiple language editions with all adequate clinimetric properties. The internal consistency of PAM13, as measured by Cronbach’s alpha, was 0.85 in this study.

#### Quality of Life

Quality of life data was collected using the Atrial Fibrillation Effect on Quality-of-Life developed by Spertus (Spertus et al., 2011) in 2011. This study specifically used the Chinese version of Zhang (Zhang et al., 2020). Twenty questions were evaluated by four dimensions (symptoms, treatment concern, daily activities, and treatment satisfaction). Responses were recorded using a 7-point Likert scale. The total score of AFEQT ranging from 0 to 100 covers the sum of the first three dimensions. The question 19, 20, and 21 regarding satisfaction with treatment and health care providers are not included in the overall AFEQT score and are each calculated independently. In the present study, the internal consistency had a Cronbach’s alpha of 0.81 for symptoms, 0.90 for treatment concern, and 0.92 for daily activities.

#### Data Collection

All the investigators accepted the uniform training and instruction before the survey. Face to face questionnaire survey was performed. Participants were informed about the study aim and study procedures. Participants filled out the questionnaire independently. Written informed consent was obtained from patients or family members.

#### Statistical Analysis

Data were skewed in the survey instruments except for the total AFEQT score. The descriptive statistics were presented using the median with interquartile range (IQR), mean and standard deviation. Independent-sample *t*-test or one-way ANOVA were used to determine the association of the categorical variables with AFEQT. Finally, multiple linear regression was constructed to explore explanatory factors for AF-related QoL.

Continuous variables were compared with a Kruskal-Wallis rank sum test and multiple pairwise comparisons were made by the using the Dwass-Steele-Critchlow- Fligner method. *P*-values lower than 0.05 were considered statistically significant. All data analyses were performed by using IBM SPSS version 25.0.

## Results

### Characteristics Description

The sample consisted of 190 patients with AF. The mean age (±SD) was 59.66 ± 9.86 years, ranging from 29 to 86 years. The majority of our participants were males (*n* = 131, 69.5%). 135 (72.1%) participants experienced education beyond high school. About 25.3% of the respondents had monthly incomes ranging from 2,000 to 4,000 yuan, and more than a half participants with a monthly income of 4,000 yuan or more. Around a third of the participants received rigorous aerobic fitness tests and personalized exercise prescriptions and took an active part in the home-based CR. The sociodemographic characteristics of the participants are shown in Table 1.

**TABLE 1:** General characteristics (*n* = 190).

**Variables**	***n***	**%**	**Mean ± SD**
**Age (years)**			59.66 ± 9.86
<45	15	7.9	
45∼60	78	41.1	
>60	97	51.1	
**Gender**			
Male	132	69.5	
Female	58	30.5	
**Education level**			
Primary school and below	16	8.4	
Middle school	37	19.5	
High school/secondary technical school	41	21.6	
Junior college	41	21.6	
University and above	55	28.9	
**Marital status**			
Married	182	95.8	
Unmarried/divorced	3	1.6	
Widowed	5	2.6	
**Work status**			
Working	58	30.5	
Retired	110	57.9	
Not working	22	11.6	
**Health insurance**			
Medical insurance for urban employees	120	63.2	
Medical insurance for non-working urban residents	39	20.5	
The new rural endowment insurance	31	16.3	
**Income monthly**			
<2000 CNY	38	20.0	
2000∼4000 CNY	48	25.3	
>4000 CNY	104	54.7	
**AF type**			
Paroxysmal AF	138	72.6	
Persistent AF	50	26.3	
Permanent AF	2	1.10	
**The number of RFCA**			
Before the first RFCA	40	21.1	
After the first RFCA	111	58.4	
After two or more RFCA	39	20.6	
**Participate in CR or not**			
Yes	62	32.6	
No	128	67.4	

*RFCA, radiofrequency catheter ablation; CR, cardiac rehabilitation.*

### Quality of Life and Patient Activation Level in Atrial Fibrillation Patients

Table 2 presents the AFEQT scores and PA levels. The total AFEQT score was 69.32 (SD:14.52), the median symptom score was 70.83 (IQR:16.7, range: 16.7–100.0), the median treatment concern score was 72.22 (IQR:22.2, range: 27.8–100.0), the median daily activities was 68.75 (IQR:17.2, range: 10.4–100.0), and the median treatment satisfaction score was 50.00 (IQR:35.4, range: 0–100). The median PAM score was 58.1 (IQR: 14.5, range: 40.7–100.0). Most participants reported activation level 3 (47.4%), followed by activation level 2 (34.2%), activation level 4 (13.7%) and activation level 1 (4.7%).

**TABLE 2:** Description of AFEQT total and subdimensions score and PA levels for the sample.

**AFEQT sub-scale**	**Mean (SD)**	**Median (Q1, Q3)**	**Number**
Symptom	70.94 (15.28)	70.83 (62.50, 79.17)	190
Treatment concern	71.59 (15.28)	72.22 (61.11, 83.33)	190
Daily activities	66.64 (17.17)	68.75 (59.90, 77.08)	190
Treatment satisfaction	56.40 (23.05)	50.00 (41.67, 75.00)	190
AFEQT-total	69.32 (14.52)	69.44 (60.88, 78.70)	190

**PAM level**	**PAM mean (SD)**	**PAM median (Q1, Q3)**	**Number (%) at level**

Level 1	43.76 (1.92)	43.70	4 (4.7)
Level 2	51.37 (1.12)	51.00	66 (34.7)
Level 3	61.64 (4.39)	60.60	90 (47.4)
Level 4	81.91 (9.27)	80.90	25 (13.2)
Total	59.89 (11.13)	58.10	190

### Associations Between Patient Activation, Sociodemographic Characteristics and Quality of Life

#### Univariate Analysis

Table 3 shows that some sociodemographic characteristics of the participants are significant. Education level, work status, healthcare insurance, monthly income, participation in CR or not, and total PA score were all found to be independently correlated with AFEQT (*p* < 0.05).

**TABLE 3:** Univariate analysis of variables against AFEQT (*n* = 190).

**Variables**	***b* (SE)**	***t*-value/*F*-value**	***p***
**Age (years)**		*F* = 0.474	0.623^b^
<45	−		
45∼60	−1.71(4.11)		
>60	0.43(4.05)		
**Gender**		*t* = 1.836	0.068^a^
Male	−		
Female	4.13(2.29)		
**Education level**		*F* = 2.490	0.045^b^
Primary school and below	−		
Middle school	0.98(4.57)		
High school/secondary technical school	−7.22(4.66)		
Junior college	−7.22(4.73)		
University and above	−4.68(4.56)		
**Marital status**		*F* = 2.680	0.071^b^
Married	−		
Unmarried/divorced	10.26(5.56)		
Widowed	12.98(7.68)		
**Work status**		*F* = 7.262	0.001^b^
Working	−		
Retired	4.34(2.29)		
Not working	13.37(3.43)		
**Health insurance**		*F* = 5.491	0.005^b^
Medical insurance for urban employees	−		
Medical insurance for non-working urban residents	7.62(2.88)		
The new rural endowment insurance	6.26(2.38)		
**Income monthly**		*F* = 8.392	< 0.001^b^
<2000 CNY	−		
2000∼4000 CNY	−8.03(2.88)		
>4000 CNY	−10.86(2.67)		
**AF type**		*F* = 0.424	0.655^b^
Paroxysmal AF	−		
Persistent AF	0.86(2.53)		
permanent AF	9.01(9.80)		
**The number of RFCA**		*F* = 1.825	0.164^b^
Before the first RFCA	−		
After the first RFCA	−3.64(2.56)		
After two or more RFCA	0.79(3.18)		
**Participate in CR or not**		*t* = 2.623	0.009^a^
Yes	−		
No	5.55(2.21)		
PAM score		*r* = 0.295	< 0.001^c^

*^a^*t*-test. ^*b*^ One-way ANOVA. ^*c*^ Spearman correlation analysis.*

#### Multiple Linear Regression Analysis

The resulting regression model (*F* = 12.968, *p* < 0.001) contained three predictors that together accounted for over 16% of the variance (adjusted *R*^2^ = 0.160) in AFEQT scores among the sample. As reported in Table 4, patient activation (β = 0.270, *p* < 0.001) contributed most to explaining the variance in AFEQT, followed by work status (β = −0.2053, *p* = 0.003) and CR (β = 0.183, *p* = 0.007).

**TABLE 4:** Stepwise multiple linear regression model of predictors of AFEQT.

**Variables**	**B (Standard error)**	**95% Confidence interval**	**β**	***t***	***p***
Constant	55.632(6.502)	(42.805, 68.459)		8.556	< 0.001
PAM score	0.341(0.086)	(0.171, 0.511)	0.270	3.963	< 0.001
Work status	−4.789(1.590)	(−7.925, −1.652)	–0.205	–3.012	0.003
CR	5.652(2.061)	(1.587, 9.718)	0.183	2.743	0.007

**F* = 12.968, *P* < 0.001, *R*^2^ = 0.173; adjusted *R*^2^ = 0.160.*

*B, unstandardized coefficients; β, standardized coefficients; CR, cardiac rehabilitation.*

#### The Influence of Patient Activation Level on Quality of Life and Its Subdimensions

We found that the symptom score was lower in level 2 compared to level 4 patients (*P* = 0.049) and lower in level 3 compared to level 4 (*P* = 0.024). Similarly, we also observed a difference in levels for the treatment concern sub-dimension. Patients in level 1 scored lower compared to those in level 4 (*P* = 0.011). Daily Activity sub-dimension also differed among levels (*P* < 0.001), with level 4 patients reporting significantly higher score than level 1 patients (*P* = 0.011) and level 2 patients (*P* = 0.002), meanwhile, level 3 patients higher than level 1 (*P* = 0.029). Otherwise, there were no significant differences in treatment satisfaction subdimension score (Table 5). Overall, the AFEQT score was lower in level 1 than level 4 patients (*P* = 0.003) and lower in level 2 than level 4 (*P* = 0.011).

**TABLE 5:** AFEQT and subdimensions across PA levels.

	**Level 1 (*n* = 9)**	**Level 2 (*n* = 66)**	**Level 3 (*n* = 90)**	**Level 4 (*n* = 25)**	***P*-value**
Symptoms					0.024^a^
Median (Q1, Q3)	62.50(43.75,72.92)	66.67(58.33,81.25)	70.83(62.50,79.17)	79.17(70.83,88.54)	
Mean (SD)	56.94(20.41)	70.39(14.99)	70.14(14.42)	79.97(12.53)	
Treatment concern					0.020^a^
Median (Q1, Q3)	58.33(44.44,70.83)	72.22(59.72,80.56)	69.44(61.11,81.25)	81.94(66.67,94.44)	
Mean (SD)	58.02(14.19)	69.66(17.02)	71.88(15.98)	80.88(13.39)	
Daily activities					< 0.001^a^
Median (Q1, Q3)	60.42(35.42,65.63)	66.67(56.25,75.00)	68.75(58.33,79.17)	77.08(64.58,90.10)	
Mean (SD)	50.00(17.86)	65.54(15.08)	66.18(17.49)	77.48(14.53)	
Treatment satisfaction					0.545^a^
Median (Q1, Q3)	50.00(37.50,75.00)	50.00(33.33,66.67)	54.17(50.00,70.08)	66.67(47.92,83.33)	
eMan (SD)	56.48(19.44)	53.72(19.98)	57.69(23.71)	60.58(30.05)	
AFEQT-total					0.001^b^
Median (Q1, Q3)	49.07(42.59,66.67)	67.59(58.80,75.93)	70.37(60.88,78.70)	77.31(69.68,89.35)	
Mean (SD)	54.22(13.02)	67.99(13.38)	68.96(14.44)	79.17(12.32)	

*^a^ Kruskal–Wallis test. ^*b*^ One-way ANOVA.*

## Discussion

This study assessed the current quality of life living with atrial fibrillation, determined the association of AF-related QoL and patient activation levels, sociodemographic characteristics. To the best of our knowledge, this is the first study focusing on this particular issue of secondary prevention in primary health care among AF patients.

### Quality of Life and Patient Activation in Patients With Atrial Fibrillation

Quality of life is an extension of mental health that reflects a comprehensive the physiological, psychological, spiritual, and social state of a person (Wang et al., 2020), an important index to measure the success of therapy. The results of our analysis revealed a moderate level of AFEQT (69.32 ± 14.52). A web-based, multicenter United States study of 295 AF patients (Guhl et al., 2019) showed that the AFEQT score was (76.3 ± 17.8), which is similar to our finding. However, the treatment satisfaction subdimension score in our sample was lower. Two possible explanations could account for this result. First, the majority of study subjects were perioperative radiofrequency catheter ablation (RFCA) inpatients with AF. A portion of the population only underwent preoperative evaluation. In addition, AF recurrences in the early postoperative period often occur due to the surgical procedure (Mujović et al., 2017), which did not perceive the postoperative improvement with persistent impaired exercise capacity (Bao et al., 2020). Second, at least 20.6% of patients had two or more history of RFCA. In a single-center prospective study, one-fifth of AF patients similarly reported a high treatment burden (Potpara et al., 2020). In patients with repeated RFCA treatment, a large decrease in expectations of RFCA and patients’ activity could question the sustainability of their treatment.

Furthermore, there were 47.4% patients in activation Levels 3 and 13.2% in Level 4. To our knowledge, there were only few articles describes PA levels in AF patients. McCabe et al. (2018) reported there were 38 and 46% were at Levels 4 and 3, respectively. The proportion of patients in level 4 was higher compared to our finding. A study regarding the distribution of activation levels in hospitalized HF patients showed that only 3% were identified as Level 4 and 40% as Level 3 (Dunlay et al., 2017). This observation might be led by the reinforcement of “sick-role” and the dependent on doctors and nurses in hospital stay. The mean (SD) PAM score in our sample was 59.89 (11.13), which was comparable to the studies regarding other chronic diseases, ranging from 51.4 (10.0) in Hendriks and Rademakers (2014) and 55.3 (11.0) in Bos-Touwen et al. (2015).

### Factors Associated With Atrial Fibrillation-Related Quality of Life

#### Sociodemographic Variables Associated With Quality of Life

The multiple linear regression demonstrated that work status was a potential factor that affect the AF-related QoL. However, there were limited research in exploring the association of work status and patient-related QoL. In the present study, patients without routine work or retired usually had higher AFEQT score and perceived their health as good. It has been identified that job tension is a known stress reaction resource, which relate to various health conditions and symptoms (Chang et al., 2006). A European Working Conditions Survey (EWCS) indicated that AF patients were more susceptible to depression attributable to job strain in Europe (Niedhammer et al., 2021). Depression and cardiovascular disease can serve as both cause and effect to each other (Lichtman et al., 2014; Tully et al., 2016). More importantly, depression can have a detrimental effect on the quality of life in AF patients. Having returned to work means regression from disease condition to an active social life, which is an important component of quality of life (Du et al., 2020). Although, the association between having returned to work and AF-related QoL has not been confirmed, these results suggest a future investigation in this aspect.

Recently, exercise-based CR has become part of the standard care for patients with cardiovascular disease. Meanwhile, interventions aimed at lifestyle and risk factors should be central in managing patients with AF. Exercise-based CR has been confirmed excellent results for AF patients, including improving AF symptoms, cardiac function and QoL, decreasing resting heart rate, increasing exercise capacity (Malmo et al., 2016; Risom et al., 2017; Smart et al., 2018; Kato et al., 2019). In our study, 32.6% participated in the home-based, patient-tailored and mobile application-guided cardiac telerehabilitation program (Cai et al., 2021), reporting higher AFEQT score than those who did not. These results were concordant with the findings of previous studies. It is also important to note that adherence to exercise training was continued even after 3 months. Hence, longer-term adherence to exercise is critical for the sustained exercise benefits. According to the literature, a comprehensive multidisciplinary CR program for AF patients treated with catheter ablation found sustained improvements with respect to physical capacity and anxiety compared to usual care at 12–24 months, without reporting changes in QoL, however (Risom et al., 2020). Future researches should focus on relationships between adherence to exercise rehabilitation and AF-related QoL, especially on patients with rehabilitation at home. With the implementation of China’s health care reform policy, a combination of medical care and nursing care and coordination of home-community-institution service system become more mature. The rehabilitation treatment and health intervention will be delivered in the community or at home.

Spearman correlation analysis revealed that the PAM score was positively related to AFEQT score. The results of multiple linear regression demonstrated that PA was a strong, independent predictor of AF-related QoL. Previous studies toward patient activation were mostly on the management of cancer (Lemanska et al., 2021) and chronic disease, such as diabetes (Kim, 2021), kidney disease (Lightfoot et al., 2021; Wilkinson et al., 2021). Regarding PA and self-management in heart failure and coronary heart disease, Liu (2018) and Li and Yao (2018) both reported that the higher the PA level, the better the disease self-management of patients. These results implicated that promoting the PA level could improve patient self-management behaviors. Similar to the reported by Zhou et al. (2020) including 287 patients with coronary heart disease, results demonstrated that the main contributions of PA to QoL were three-fold as follows: physical health, mental health and specific functional status.

### Activation Levels Associated With Quality of Life

Our analysis indicated that subsets in symptoms, daily activities, treatment concerns and also the total AFEQT score differed among the levels. As expected, patients in Level 4 reported higher symptoms score than those from other levels. Individuals actively engaged in health management may perceive less symptoms than those did not (Sears et al., 2005). These findings highlighted the importance of integrating evaluation of symptoms and symptomatic burden into routine care for AF patients in different activation levels, facilitating internal or external social resources to use effectively, and fostering progression of PA levels and improvement of symptoms. The positive psychological interventions have developed and gained great attention. Särnholm et al. (2017) substantiated the efficacy of a novel cognitive behavioral therapy (CBT, exposure-based Therapy combined with education and Behavioral activation) approach to reduce symptoms and improve QoL in AF patients.

A Mixed study (Stridsman et al., 2019) reported the inability to engage in daily activities and reduced exercise capacity due to AF symptoms. However, the relationship between activation level and daily activity has not been widely studied. We did observe that there was no significant difference of the median daily activity score across four levels in our study. In actual practice, patients who participated in CR often independent the severity of the symptoms. Moreover, engaging in routine physical activities may be linked to a decreased burden in managing daily household and social activities.

In the treatment concern sub-dimension measure patients’ medical knowledge has a powerful influence on patient-perceived treatment concern, which includes the patients’ perception of specific treatment side-effects and the negative influences on QoL (Eton et al., 2012). Low disease and treatment-related knowledge in lower activation levels (Level 1 and Level 2) contribute to a lack of informed decision-making in AF patients. These results suggested that the patient’s experience and concern should be shared in-depth during physician-patient consultations. Positive computing technology is a well-explored and growing priority in both positive psychology and intelligent healthcare (Yarosh and Schueller, 2017). Magnani et al. (2017) reported that smartphone-based relational agent intervention positively affected favorable improvements in QoL and self-reported medication adherence. AF education, common symptoms, adherence challenges, and patient activation formed the relational agent’s dialogue content. Findings also showed that there were no significant association between PA level and treatment satisfaction. Further study should pay more attention on the relationship between satisfaction with medical treatment and PA across samples.

This study has certain limitations. First, the population in the study was obtained from a Grade A hospital in Nanjing, which caused a certain degree of selective bias. Second, considerable gaps are existing in outcomes of treatment satisfaction compared to other studies. It is worthy of thorough exploring and investigation. Finally, this research only included few sociodemographic characteristics and clinical variables associated with the life quality in AF patients. However, the main objective of this study was to investigate the positive relationships between PA and QoL, future research is warranted to delve deeper into the correlation of other risk factors and QoL, PA and risk factors management.

## Conclusion

The level of QoL of patients with AF was generally moderate. Among the influencing factors such as education level, work status, healthcare insurance, monthly income, participation in CR or not, PA was the most important factor for QoL. The influences of PA level on subdimensions of AF-related QoL were symptoms, daily activities, and treatment concern. Given the importance of PA for QoL, more attention should be paid to the PA of AF patients in the future, including the study on developing tailor-made interventions aimed at the activation level.

## Data Availability Statement

The raw data supporting the conclusions of this article will be made available by the authors, without undue reservation.

## Ethics Statement

The studies involving human participants were reviewed and approved by the Ethics Committee of The First Affiliated Hospital of Nanjing Medical University (Approval No. 2021-NT-36). The patients/participants provided their written informed consent to participate in this study.

## Author Contributions

JW, QW, GS, ZB, MG, YP, LW, and GY contributed to the study design. JW, QW, ZB, SL, and TY contributed to the data collection and data analysis. JW, QW, and MG manuscript contributed to the writing. All authors contributed to manuscript revision and approval of final submission.

## Conflict of Interest

The authors declare that the research was conducted in the absence of any commercial or financial relationships that could be construed as a potential conflict of interest.

## Publisher’s Note

All claims expressed in this article are solely those of the authors and do not necessarily represent those of their affiliated organizations, or those of the publisher, the editors and the reviewers. Any product that may be evaluated in this article, or claim that may be made by its manufacturer, is not guaranteed or endorsed by the publisher.
